# Strongly frustrated triangular spin lattice emerging from triplet dimer formation in honeycomb Li_2_IrO_3_

**DOI:** 10.1038/ncomms10273

**Published:** 2016-01-18

**Authors:** Satoshi Nishimoto, Vamshi M. Katukuri, Viktor Yushankhai, Hermann Stoll, Ulrich K. Rößler, Liviu Hozoi, Ioannis Rousochatzakis, Jeroen van den Brink

**Affiliations:** 1 Institute for Theoretical Solid State Physics, IFW Dresden, Helmholtzstrasse 20, 01069 Dresden, Germany; 2 Joint Institute for Nuclear Research, Joliot-Curie 6, 141980 Dubna, Russia; 3 Max-Planck-Institut für Physik komplexer Systeme, Nöthnitzer Street 38, 01187 Dresden, Germany; 4 Institute for Theoretical Chemistry, Universität Stuttgart, Pfaffenwaldring 55, 70550 Stuttgart, Germany; 5 Department of Physics, Technical University Dresden, Helmholtzstrasse 10, 01069 Dresden, Germany

## Abstract

Iridium oxides with a honeycomb lattice have been identified as platforms for the much anticipated Kitaev topological spin liquid: the spin-orbit entangled states of Ir^4+^ in principle generate precisely the required type of anisotropic exchange. However, other magnetic couplings can drive the system away from the spin-liquid phase. With this in mind, here we disentangle the different magnetic interactions in Li_2_IrO_3_, a honeycomb iridate with two crystallographically inequivalent sets of adjacent Ir sites. Our *ab initio* many-body calculations show that, while both Heisenberg and Kitaev nearest-neighbour couplings are present, on one set of Ir–Ir bonds the former dominates, resulting in the formation of spin-triplet dimers. The triplet dimers frame a strongly frustrated triangular lattice and by exact cluster diagonalization we show that they remain protected in a wide region of the phase diagram.

## Abstract (web summary)

Honeycomb iridates are candidates for the realization of a Kitaev spin liquid, displaying properties such as the protection of quantum information. Here, the authors investigate the phase diagram of honeycomb Li_2_IrO_3_, using quantum chemistry methods to derive the relevant effective spin Hamiltonian.

## Graphical abstract

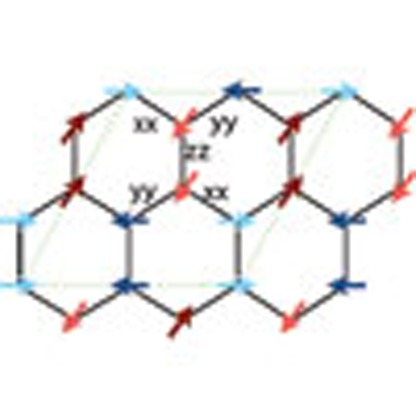

As early as in the 1970s it was suggested that quantum spins in a solid can, instead of ordering in a certain pattern, form a fluid type of ground state—a quantum spin liquid^1^,^2^. Theory predicts a remarkable set of collective phenomena to occur in spin liquids^3^. In the honeycomb lattice Kitaev spin model^4^, for instance, a spin-liquid state that has different topological phases with elementary excitations displaying Majorana statistics has been anticipated. This has been argued to be relevant for applications in topological quantum computing^5^,^6^,^7^,^8^,^9^.

The essential feature of the Kitaev model is that there is a different type of spin coupling for each of the three magnetic bonds originating from a given *S*=1/2 spin site, 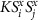, 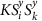 and 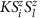, where *j*, *k* and *l* are *S*=1/2 nearest neighbours (NN's) of the reference site *i* and *K* is the Kitaev coupling strength. However, finding materials in which the Kitaev spin model and the spin-liquid ground state are realized has proven to be very challenging^3^. In this respect the strongly spin-orbit coupled honeycomb iridates have recently been brought to the fore^10^,^11^. These compounds have the chemical formula *A*_2_IrO_3_, with *A*=Na or Li, and contain Ir^4+^ ions in the centre of oxygen octahedra that form a planar hexagonal network. Each Ir^4+^ ion has five electrons in the 5*d* shell which the crystal field splits into a *t*_2*g*_ and an *e*_*g*_ manifold. Since the crystal field splitting is large, the lowest-energy electron configuration is 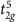. This is equivalent to the *t*_2*g*_ shell containing a single hole with spin *S*=1/2. However, the 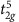 state additionally bears a finite effective angular moment *L*_eff_=1. The strong spin-orbit coupling for 5*d* electrons therefore splits up the 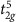 manifold into an effective total angular momentum 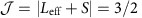 quartet and a 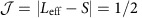 doublet. As for the hole the latter is lowest in energy, an effective spin 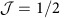 doublet (often referred to as a pseudospin 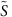) defines to first approximation the local ground state of the Ir^4+^ ion.

Whereas the formation of such a local 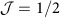 doublet is well-known for Ir^4+^ ions inside an undistorted oxygen octahedron^12^, the remarkable insight of refs 10, 11 is that when two such octahedra share an edge, the magnetic superexchange interactions between the 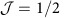 sites are in principle precisely of Kitaev type. This observation has made the *A*_2_IrO_3_ honeycomb iridates prime candidate materials in the search for Kitaev spin-liquid ground states.

Experimentally, however, both Na_2_IrO_3_ and Li_2_IrO_3_ have been found to order magnetically below 15 K (refs 13, 14). While inelastic neutron scattering^15^, X-ray diffraction^16^ and resonant inelastic X-ray scattering experiments^17^ indicate an antiferromagnetic (AF) zigzag ordering pattern in Na_2_IrO_3_, the nature of the magnetic order of Li_2_IrO_3_ is to date unknown^13^,^14^. The questions that arise are therefore, (i) which magnetic instability preempts the formation of the spin-liquid state, and how close does the system remains to that state.

To answer these fundamental questions it is essential to quantify the relative strengths of the NN magnetic interactions in Li_2_IrO_3_, which are already known to be not only of Kitaev, but also of Heisenberg type. The observed zigzag order in its counterpart system Na_2_IrO_3_ has indeed been rationalized on the basis of ferromagnetic (FM) Heisenberg *J* and AF Kitaev *K* couplings^18^,^19^,^20^, but also interpreted in terms of an AF *J* and FM *K* (refs 13, 15, 21, 22). Recent *ab initio* many-body calculations favour the latter scenario, with a relatively large FM Kitaev exchange and significantly weaker AF NN Heisenberg interactions in this material^23^. This scenario is also supported by investigations of model Hamiltonians derived by downfolding schemes based on density functional theory calculations^24^. Besides the NN terms, strongly frustrating longer range exchange couplings involving the second (*J*_2_) and third (*J*_3_) iridium coordination shells were also shown to be relevant^13^,^15^,^20^, resulting in very rich magnetic phase diagrams^13^,^23^,^25^.

On the basis of the similarity in crystal structure, one might naively expect that the magnetic interactions in *A*=Li are similar to the ones in *A*=Na. Here we show that this is not at all the case. The strengths of the NN interactions *J* and *K* turn out to crucially depend on the Ir–O–Ir bond angles and distances. Employing *ab initio* wave-function quantum chemistry methods, we find in particular that in contrast to Na_2_IrO_3_ (ref. 23) the Heisenberg coupling *J* in Li_2_IrO_3_ even has opposite signs for the two crystallographically inequivalent sets of adjacent Ir sites. This behaviour follows a general trend of *J* and *K* as functions of bond angles and interatomic distances that we have established through a larger, additional set of quantum chemistry calculations. The latter show that the NN Heisenberg *J* has a parabolic dependence on the Ir–O–Ir bond angle and at around 98° changes sign. This explains why in Na_2_IrO_3_, with Ir–O–Ir angles in the range of 98–100° (ref. 15), all *J*′s are positive, while in Li_2_IrO_3_, which has significantly smaller bond angles ∼95°(ref. 26), the FM component to the NN Heisenberg exchange is much stronger. The large FM coupling 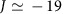 meV on one set of Ir–Ir links in Li_2_IrO_3_ gives rise to an effective picture of triplet dimers composing a triangular lattice. To determine the magnetic phase diagram as a function of the strength of the second and third neighbour exchange interactions (*J*_2_ and *J*_3_) we use for this effective triplet-dimer model a semiclassical approach, which we further confront to the magnetic phase diagram for the original honeycomb Hamiltonian calculated by exact cluster diagonalization. This comparison shows that indeed the triplet dimers act as rigid objects in a wide range of the *J*_2_–*J*_3_ parameter space. We localize Li_2_IrO_3_ in a parameter range where the phase diagram has incommensurate magnetic order, the nature of which goes beyond the standard flat helix modulation scenario, owing to the Kitaev exchange anisotropy.

## Results

### Heisenberg–Kitaev Hamiltonian

**Figure 1:**
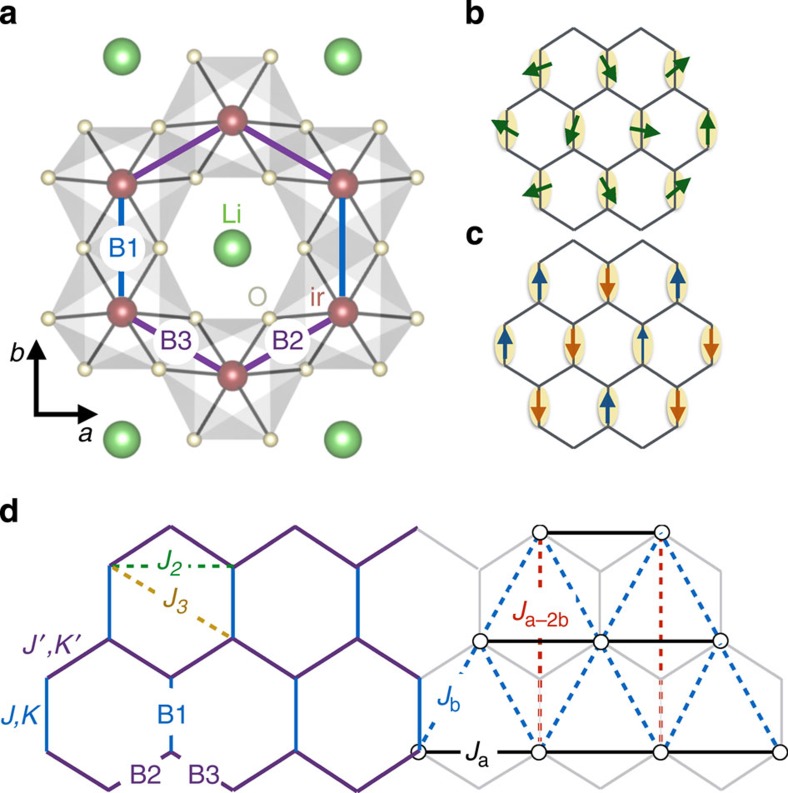
Honeycomb structure of Li_2_IrO_3_ and mapping onto an effective triangular lattice of triplet spins. (**a**) The two distinct sets of NN links^26^ are labelled as B1 (along the crystallographic *b* axis) and B2/B3. (**b**) The large FM interaction *J*=−19.2 meV on B1 bonds stabilizes rigid *T*=1 triplets that frame an effective triangular lattice. The triplet dimers remain protected in a wide region of the phase diagram, including the incommensurate ICx and (**c**) diagonal-zigzag phase, see text. (**d**) Representative exchange couplings for B1 (*J*, *K*), B2/B3 (*J*′, *K*′), second neighbour (*J*_2_) and third neighbour (*J*_3_) paths on the original hexagonal grid are shown. *J*_***δ***_ (***δ***∈{**a**, **b**, **a**−2**b**}) are isotropic exchange interactions on the effective triangular net.

The experimental data reported in ref. 26 indicate *C*_2*h*_ point-group symmetry for one set of NN IrO_6_ octahedra, denoted as B1 in Fig. 1, and slight distortions of the Ir_2_O_2_ plaquettes that lower the symmetry to *C*_*i*_ for the other type of adjacent octahedra, labelled B2 and B3. The most general, symmetry allowed form of the effective spin Hamiltonian for a pair of NN Ir *d*^5^ sites, as discussed in Methods and Supplementary Note 1, is then


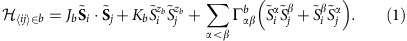


The *b* index refers to the type of Ir–Ir link (*b*∈{B1,B2,B3}). Whereas the Hamiltonians 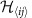 on the Ir–Ir links B2 and B3 are related by symmetry, the bond B1 is distinct from a symmetry point of view. Further, 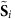 and 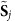 denote pseudospin-1/2 operators, *J*_*b*_ is the isotropic Heisenberg interaction and *K*_*b*_ the Kitaev coupling. The latter plus the off-diagonal coefficients 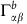 define the symmetric anisotropic exchange tensor. It is shown below that these 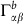 elements are not at all negligible, as assumed in the plain Kitaev–Heisenberg Hamiltonian.

In equation (1), *α* and *β* stand for components in the local, Kitaev bond reference frame {**x**_*b*_, **y**_*b*_, **z**_*b*_}^10^. The **z**_*b*_ axis is perpendicular to the Ir_2_O_2_ plaquette (Methods section, Supplementary Note 2 and Supplementary Fig. 1). In the following, we denote *J*_*B*1_=*J*, *J*_*B*2_=*J*_*B*3_=*J*′, *K*_*B*1_=*K*, *K*_*B*2_=*K*_*B*3_=*K*′ and similarly for the 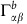 elements.

### NN exchange interactions

To make reliable predictions for the signs and strengths of the exchange coupling parameters we rely on many-body quantum chemistry machinery, in particular, multireference configuration interaction (MRCI) computations^27^ on properly embedded clusters. Multiconfiguration reference wave functions were first generated by complete active space self-consistent field (CASSCF) calculations. For two NN IrO_6_ octahedra, the finite set of Slater determinants was defined in the CASSCF treatment in terms of ten electrons and six (Ir *t*_2*g*_) orbitals. The self-consistent field optimization was carried out for an average of the lowest nine singlet and nine triplet states associated with this manifold. All these states entered the spin-orbit calculations, both at the CASSCF and MRCI levels. On top of the CASSCF reference, the MRCI expansion additionally includes single and double excitations from the Ir *t*_2*g*_ shells and the 2*p* orbitals of the bridging ligands. Results in good agreement with the experimental data were recently obtained with this computational approach for related 5*d*^5^ iridates displaying corner-sharing IrO_6_ octahedra^28^,^29^,^30^.

Relative energies for the four low-lying states describing the magnetic spectrum of two NN octahedra and the resulting effective coupling constants are provided in Table 1. To derive the latter, we map the quantum chemically computed eigenvalues listed in the table to the eigenvalues of the effective magnetic Hamiltonian in equation (1). For the effective picture of 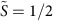 pseudospins assumed in equation (1), the set of four eigenfunctions contains the singlet 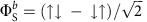 and the triplet components 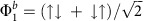, 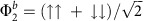, 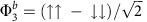. In *C*_2*h*_ symmetry, the ‘full' spin-orbit wave functions associated to 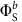, 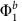, 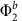 and 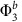 transform according to the *A*_*g*_, *B*_*u*_, *B*_*u*_ and *A*_*u*_ irreducible representations, respectively. Since two of the triplet terms may interact, the most compact way to express the eigenstates of the effective Hamiltonian in equation (1) is then 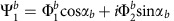, 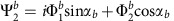, 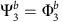 and 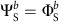. The angle *α*_*b*_ parametrizes the amount of 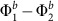 mixing, related to finite off-diagonal 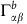 couplings. This degree of admixture is determined by analysis of the full quantum chemistry spin-orbit wave functions. The effective parameters provided in Table 1 are obtained for each type of Ir–Ir link by using the 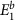, 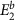, 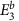, 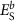 MRCI relative energies and the 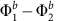 mixing coefficients (see Methods and Supplementary Note 1). For a comparison of the effective parameters derived from CASSCF and MRCI relative energies, see Supplementary Tables 1 and 2.

**Figure 2:**
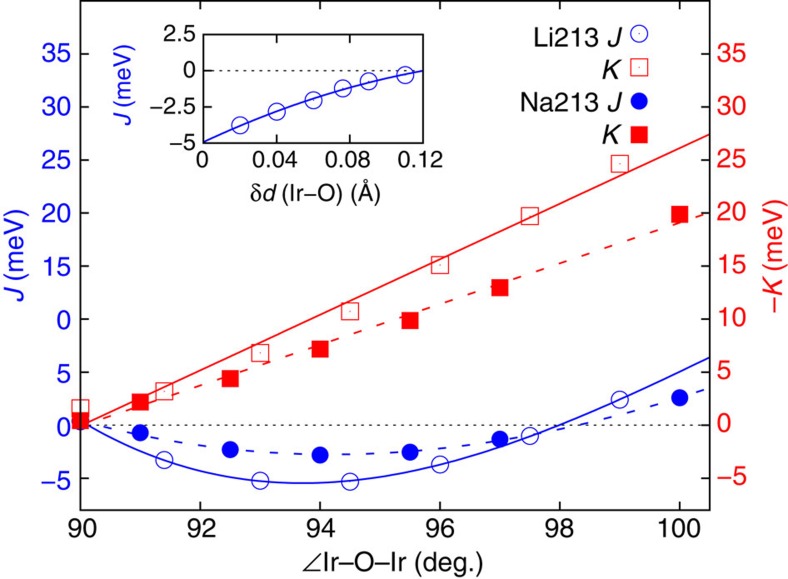
Variation of the Heisenberg and Kitaev exchange couplings with the Ir–O–Ir angle in idealized honeycomb structural models. Results of spin-orbit MRCI calculations are shown, for NN Ir–Ir links in both Li213 (continuous lines) and Na213 (dashed). For each system, the NN Ir–Ir distances are set to the average value in the experimental crystal structure^15^,^26^ and the Ir–O bond lengths are all the same. Consequently, *J*=*J*′ and *K*=*K*′. The variation of the Ir–O–Ir angles is the result of gradual trigonal compression. Note that 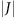, 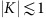 meV at 90°. Inset: dependence of the NN *J* in Li213 when the bridging O's are gradually shifted in opposite senses parallel to the Ir–Ir axis.

For the B1 links in Li_2_IrO_3_ (Li213) we find that both *J* and *K* are FM, in contrast to Na_2_IrO_3_ (Na213) where *J* is AF for all pairs of Ir NN's^23^. Insights into this difference between the Li and Na iridates are provided by the curves plotted in Fig. 2, displaying the dependence of the NN *J* on the amount of trigonal distortion for simplified structural models of both Li213 and Na213. The trigonal compression of the O octahedra translates into Ir–O–Ir bond angles >90°. Additional distortions giving rise to unequal Ir–O bond lengths, see the footnotes in Table 1, were not considered in these idealized lattice configurations. Interestingly, we find that for 90° bond angle—the case for which most of the superexchange models are constructed^10^,^11^,^18^,^22^—both *J* and *K* are very small, 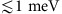.

In Fig. 2, while |K| monotonously increases with the Ir–O–Ir bond angle, *J* displays a parabolic behaviour and with a minimum at ∼94°. Indeed on the basis of simplified superexchange models one expects *J* to be minimal at around a bond angle close to 90°. However, from superexchange models it is at the same time expected that *K* is substantial for such bond angles. The difference between the *ab initio* results for 90° Ir–O–Ir angles and the predictions of simplified superexchange models originates from assuming in the latter perfectly degenerate Ir 5*d* and O 2*p* orbitals and from the subsequent cancellation of particular intersite *d*–*p*–*d* exchange paths. The quantum chemistry calculations show that the Ir 5*d* levels are not degenerate (nor the O 2*p* functions at a given site); the symmetry lowering at the Ir/O sites and this degeneracy lifting are related to the strongly anisotropic, layered crystal structure. For the actual honeycomb lattice with trigonal distortions of oxygen cages, one should develop a superexchange theory using the trigonal 5*d* orbital basis, as well as the correspondingly oriented oxygen orbitals. This produces a more general anisotropy than the Kitaev one. This is the essential reason we find at 90° for Na213 (Ir–Ir average distances of 3.133 Å): *J*=0.32, *K*=−0.43, Γ_*xy*_=2.6, Γ_*zx*_=−1.3, Γ_*yz*_=1.3 and for Li213 (Ir–Ir average distances of 2.980 Å): *J*=0.40, *K*=−1.60, Γ_*xy*_=5.4, Γ_*zx*_=−2.8, Γ_*yz*_=2.8 meV. For both materials *K* actually turns out to be the smallest of the anisotropic exchange constants at 90°. The small value of *K* may give the impression that only a weak uniaxial anisotropy is active (Supplementary Table 3). However, if one diagonalizes the full Γ matrix to obtain its principal axes (which in general are distinct from any crystallographic directions) and corresponding anisotropies, one finds sizable anisotropic exchange constants as large as few meV.

Our investigation also shows that the large FM *J* value obtained for the B1 Ir–Ir links in Li213 is the superposition of three different effects (Fig. 2): (i) an Ir–O–Ir bond angle smaller than the value of ≈98° where *J* changes sign which in contrast to Na213 takes us into the FM regime, (ii) the shift to lower values of the minimum of the nearly parabolic *J* curve in Li213 as compared with Na213 and further (iii) the additional distortions giving rise to three different sets of Ir–O bond lengths for each IrO_6_ octahedron. The latter are significantly stronger in Li213, remove the degeneracy of the Ir *t*_2*g*_ levels and make that the NN B1 *J* is even lower than the minimum of the parabola displayed in Fig. 2. It is also interesting that the off-diagonal Γ_*yz*_ and Γ_*zx*_ couplings on B1 have about the same strength with the Kitaev *K* (Table 1). Our *ab initio* results justify more detailed model Hamiltonian investigations of such off-diagonal couplings along the lines of refs 21, 22, 24.

For the B2 and B3 links, the Ir–O bonds on the Ir–O_2_–Ir plaquette have different lengths and the symmetry of the two-octahedra block is lowered to *C*_*i*_ (ref. 26). The *ab initio* data show that consequently the FM exchange is here disfavoured such that *J*′ turns AF. This is illustrated in the inset of Fig. 2, where we plot the evolution of the NN Heisenberg coupling when in addition to trigonal distortions the bridging ligands on the Ir–O_2_–Ir plaquette are gradually shifted in opposite senses parallel to the Ir–Ir axis. For the reference equilateral plaquette, the Ir–O–Ir bond angle is set to the average value in the experimental structure, 95° (ref. 26). It is seen that such additional distortions indeed enhance the AF contribution to the Heisenberg superexchange. Although the bond symmetry is lower for the B2/B3 links, the analysis of the spin-orbit wave functions shows however negligible additional mixing effects and the *ab initio* results were still mapped onto a *C*_2*h*_ model with 
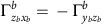
.

### Longer range interactions

Having established the dominant NN couplings we now turn to the magnetic phase diagram of Li213 including the effect of second and third neighbour Heisenberg interactions *J*_2_ and *J*_3_. The latter are known to be sizable^22^ and to significantly influence certain properties^13^,^15^,^23^,^25^. However, since correlated quantum chemistry calculations for these longer range interaction terms are computationally much too demanding, we investigate their effect by computations for extended effective Hamiltonians that use the *ab initio* NN magnetic interactions listed in Table 1 and adjustable isotropic *J*_2_, *J*_3_ exchange couplings.

### Triplet dimers

With strong FM exchange on the B1 bonds, a natural description of the system consists in replacing all B1 pairs of Ir 1/2 pseudospins by rigid triplet degrees of freedom. This mapping leads to an effective model of spin *T*=1 entities on a triangular lattice, captured by the Hamiltonian


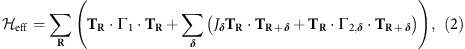


where ***δ***∈{**a**, **b**, **a**−2**b**} (Fig. 1d and Supplementary Fig. 2). It includes both on-site (**Γ**_1_) and intersite (*J*_***δ***_, **Γ**_2,***δ***_) effective interaction terms. While the explicit expressions of these terms are given in Methods, the essential features of the model are as follows. First, among the few different contributions to **Γ**_1_, there is an effective coupling of the form 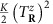. Since *K*<0, this term selects the two triplet components with *T*_*z*_=±1 and therefore acts as an easy-axis anisotropy. Second, there are two different types of effective exchange couplings between NN triplets, see Fig. 1d. This asymmetry reflects the constitutive difference between bonds B1 and B2/B3. Finally, there is also an effective longer range exchange driven by the *J*_3_ interaction in the original hexagonal model.

According to our *ab initio* results, the on-site anisotropy splitting is 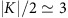 meV, about twice the ordering temperature in Li213. Naively, this may suggest a truncation of the local Hilbert space such that it includes only the *T*_*z*_=±1 components, which would lead to an effective doublet instead of a triplet description. However, such a truncation would not properly account for transverse spin fluctuations driven by intersite exchange (which may even exceed the on-site splitting, depending on the values of *J*_2_ and *J*_3_) or for the coupling to the *T*_*z*_=0 component via off-diagonal terms in **Γ**_1_. Lacking *a priori* a clear separation of energy scales, one is thus left with a description in terms of *T*=1 triplets.

**Figure 3:**
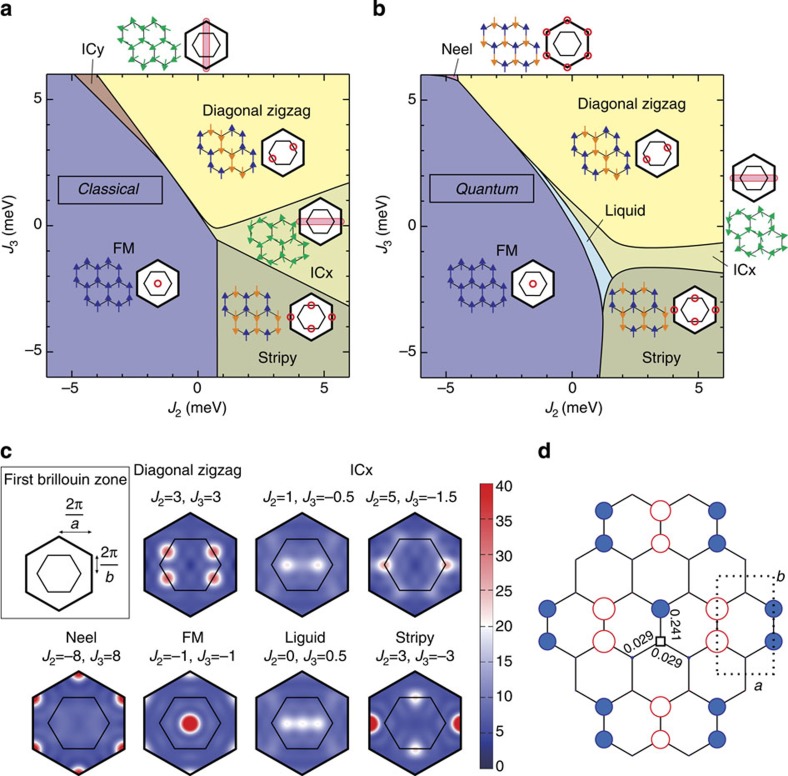
Magnetic phase diagrams and spin structure factor. Phase diagram of Li213 in the *J*_2_–*J*_3_ plane with the NN couplings listed in Table 1, along with schematic spin configurations and Bragg peak positions (red circles) for each phase. (**a**) Classical phase diagram of the effective spin *T*=1 model on the triangular lattice, found by a numerical minimization of the interaction matrix **Λ**(k) in the Brillouin zone (BZ). The actual ground-state configurations in the incommensurate regions ICx and ICy can be much richer than the standard coplanar helix states owing to anisotropy, see text. (**b**) Quantum mechanical phase diagram for the original spin-1/2 model. (**c**) Structure factor 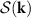 for representative momenta in different phases. Note that in the ICx phase, the peak position (±*Q*_*a*_, 0) takes values between 0<*Q*_*a*_≤2*π*/*a*, depending on *J*_2_ and *J*_3_. (**d**) Long range spin–spin correlation profiles 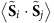 at *J*_2_=*J*_3_=3 (that is, inside the diagonal-zigzag phase), as obtained by exact diagonalization (ED) calculations. The reference Ir site is shown as a black square rectangle, positive (negative) correlations are denoted by filled blue (open red) circles whose radii scale with 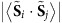. We also show explicitly the actual values for the NN correlations.

In momentum space, the effective model takes the form


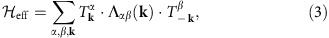


where 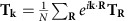, *N* is the number of B1 bonds and **Λ**(**k**) is a symmetric 3 × 3 matrix (Supplementary Note 3). Since *T*=1, the classical limit is expected to yield a rather accurate overall description of the phase diagram. The minimum eigenvalue *λ*_**Q**_ of **Λ**(**k**) over the Brillouin zone provides a lower bound for the classical ground-state energy^31^,^32^,^33^,^34^. As shown in Fig. 3a, there exist five different regions for 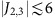 meV, three with commensurate (FM, diagonal zigzag and stripy) and two with incommensurate (IC) **Q** (we call them ICx and ICy, with **Q**=(*q*, 0) and (0, *q*), respectively). In all commensurate regions, the state 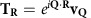 (where **v**_**Q**_ is the eigenvector associated with *λ*_**Q**_) saturates the above lower energy bound and in addition satisfies the spin length constraint |**T**_**R**_|=1 for all **R**. We note in particular that compared to the more symmetric case of Na213 (ref. 23), only the diagonal-zigzag configurations are favoured in Li213, with FM correlations along the two diagonal directions of the lattice. The third, horizontal zigzag configuration is penalized by the strong FM Heisenberg coupling on the B1 links. Correspondingly, we expect Bragg peaks only at two out of the three **M** points of the Brillouin zone, namely 
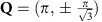
 (see 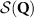 in Fig. 3c and Supplementary Fig. 3). Turning to the incommensurate regions ICx and ICy, the minimum eigenvalue *λ*_**Q**_ is nondegenerate, which implies that one cannot form a flat helical modulation that saturates the low energy bound and satisfies the spin length constraint for all **R**. Especially for ICx that is the most likely candidate for Li213 (see below), this opens the possibility for nontrivial nonplanar modulations of the magnetization.

### Exact diagonalization calculations

To establish the effect of quantum fluctuations and further test the triplet-dimer picture, we additionally carried out exact diagonalization calculations on 24-site clusters for the original honeycomb spin-1/2 model including the effect of *J*_2_ and *J*_3_. Periodic boundary conditions were applied, as in previous studies^18^,^23^. We calculated the static spin-structure factor 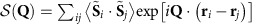 as a function of *J*_2_ and *J*_3_ while fixing the NN magnetic couplings to the ones in Table 1. For a given set of *J*_2_ and *J*_3_ values, the dominant order is determined according to the wave number **Q**=**Q**_max_ providing a maximum of 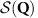. The resulting phase diagram is given in Fig. 3b. For each phase, the real-space spin configuration and the reciprocal-space Bragg peak positions are shown. In the absence of *J*_2_ and *J*_3_, the system is in a spin-liquid phase characterized by a structureless 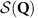 (Fig. 3c) that is adiabatically connected to the Kitaev liquid phase for 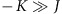 (ref. 10). By switching on *J*_2_ and *J*_3_, we recover most of the classical phases of the effective spin-1 model, including the ICx phase, albeit with a smaller stability region due to finite-size effects. That the 24-site cluster correlations do not show the ICy phase may well be an intrinsic effect, given that the classical ICy region is very narrow. We also find an AF Néel state region, which is now shifted to larger *J*_3_'s as compared with Na213 (ref. 23), due to the large negative *J* on B1 bonds.

We note that detecting the diagonal-zigzag phase by exact diagonalization calculations requires large-size setups of lattice sites. This is related to the proximity of this phase to the special point **Γ**=0 where the model is highly frustrated. Indeed, in this limit the classical ground-state manifold consists of a one-parameter family of states with two sublattices of spins with arbitrary relative orientation angle. This situation is common in various well-known frustrated models, such as the *J*_1_–*J*_2_ model on the square lattice^35^,^36^,^37^. The lifting of the accidental degeneracy, either by quantum fluctuations or due to a finite **Γ** (Supplementary Note 4, Supplementary Figs 4 and 5), and the associated locking mechanism between the two sublattices involve a very large length scale^38^,^39^. This explains why our exact spin–spin correlation profiles provided in Fig. 3d show that the two sublattices are nearly decoupled from each other.

Except for the Néel and the spin-liquid phase, all other phases feature rigid triplets on the B1 bonds. This is shown in Fig. 3d for the diagonal-zigzag phase at *J*_2_=*J*_3_=3, where the NN correlation function on the B1 bonds, 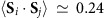, almost saturates to the full spin-triplet value of 1/4. This shows that the effective triplet picture is quite robust.

### Comparison to experiment

Our result for rigid triplet degrees of freedom finds support in recent fits of the magnetic susceptibility data, which yield effective moments of 2.22 *μ*_B_ for Li213 (ref. 40), much larger than the value of 1.74 *μ*_B_ expected for an isotropic 1/2 spin system. Triplet dimerization was earlier suggested to occur in the chain-like compound In_2_VO_5_ (ref. 41). FM, quintet dimers were also proposed to form in ZnV_2_O_4_ (ref. 42).

Turning finally to the nature of the actual magnetic ground state of Li213, we first note that the longer range couplings *J*_2_ and *J*_3_ are expected to be both AF^13^,^15^ and to feature values not larger than 5–6 meV (ref. 15) in honeycomb iridates, which suggests that Li213 orders either with a diagonal-zigzag or ICx pattern. Recent magnetic susceptibility and specific heat measurements indeed show indications (ref. 14) that the magnetic ground state of Li213 could be different from AF zigzag, while powder diffraction and inelastic neutron scattering data (R. Coldea, personal communication) show signatures of incommensurate magnetic order. These experimental findings are consistent with the ICx spin configuration. As explained above, the actual nature of this phase goes beyond the standard flat helical modulations because the latter are penalized by the anisotropic exchange terms in the Hamiltonian. It should be noted that the incommensurate type of magnetic order in Li_2_IrO_3_ has also been rationalized with model Hamiltonian calculations by including additional long range anisotropic Kitaev couplings on the honeycomb lattice^43^.

## Conclusions

To summarize, we have established a microscopic spin model and zero-temperature phase diagram for the layered honeycomb iridate Li_2_IrO_3_, one of the proposed realizations of the spin-1/2 Kitaev–Heisenberg model with strongly spin-orbit coupled Ir^4+^ magnetic ions. *Ab initio* quantum chemistry electronic-structure calculations show that, in contrast to Na_2_IrO_3_, the structural inequivalence between the two types of Ir–Ir links has a striking influence on the effective spin Hamiltonian, leading in particular to two very different nearest-neighbour superexchange pathways, one weakly antiferromagnetic 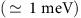 and another strongly ferromagnetic (−19 meV). The latter gives rise to rigid spin-1 triplets on a triangular lattice that remain well protected in a large parameter regime of the phase diagram, including a diagonal zigzag and an incommensurate ICx phase. In view of these theoretical findings and the experimental observation of an incommensurate magnetic propagation vector in neutron diffraction (R. Coldea, personal communication), we propose that the magnetic ground state of Li_2_IrO_3_ lies in the incommensurate ICx phase. Settling its detailed nature and properties calls for further, dedicated experimental and theoretical investigations.

## Methods

### Embedded-cluster quantum chemistry calculations

All *ab initio* calculations were carried out with the quantum chemistry package Molpro^44^. Embedded clusters consisting of two NN edge-sharing IrO_6_ octahedra were considered. To accurately describe the charge distribution at sites in the immediate neighbourhood^45^,^46^, the four adjacent Ir^4+^ ions and the closest 22 Li^+^ neighbours were also explicitly included in the actual cluster. The surrounding solid-state matrix was modeled as a finite array of point charges fitted to reproduce the crystal Madelung field in the cluster region. The spin-orbit treatment was carried out according to the procedure described in ref. 47, using spin-orbit pseudopotentials for Ir (Supplementary Note 1).

Even with trigonal distortions of the oxygen cages, the point-group symmetry of a given block of two NN IrO_6_ octahedra is *C*_2*h*_. Since the *C*_2_ axis lies here along the Ir–Ir bond, the effective magnetic Hamiltonian for two adjacent Ir sites is most conveniently expressed in a local reference system {**X**_*b*_, Y_*b*_, **Z**_*b*_} with **X**_*b*_ along the Ir–Ir link (**Z**_*b*_ is always perpendicular to the Ir_2_O_2_ plaquette). It reads


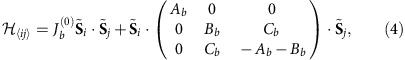


where *b*∈{B1,B2,B3}. The diagonal elements in the second term on the right hand side sum up to 0 to give a traceless symmetric anisotropic exchange tensor. If **X**_*b*_ is *C*_2_ axis, only one off-diagonal element is nonzero.

In the local Kitaev reference frame {**x**_*b*_, **y**_*b*_, **z**_*b*_}, that is rotated from {**X**_*b*_, **Y**_*b*_, **Z**_*b*_} by 45^o^ about the ***Z***_*b*_=***z***_*b*_ axis (Supplementary Note 2, Supplementary Fig. 1 and refs 10, 23), the Hamiltonian shown above in equation (4) is transformed to the Hamiltonian in equation (1). For the latter, the effective exchange couplings are obtained for each type of Ir–Ir link as


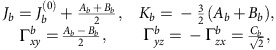


where the connection to the quantum chemically computed eigenvalues provided in Table 1 (and Supplementary Tables 1 and 2) is


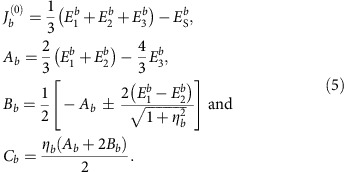



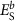
, 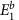, 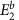, 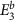 are the *ab initio* eigenvalues, 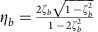 and *ζ*_*b*_=sin*α*_*b*_, where *α*_*b*_ is the mixing parameter.

### Effective spin *T*=1 description

To find the effective interactions between the B1 triplet dimers, we begin by deriving the equivalent operators in the *T*_**R**_=1 manifold for a B1 bond at position **R**, where **T**_**R**_=**S**_**R**,1_+**S**_**R**,2_ and **S**_**R**,1_, **S**_**R**,2_ are the ionic Ir pseudospins defining the B1 bond. If the projector in the *T*_**R**_=1 manifold is tagged as *P*_*T*_, we obtain for the dipolar channel 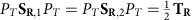, while for the quadrupolar channel


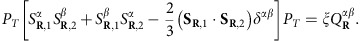



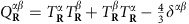
 is here the quadrupolar operator for a spin-1 degree of freedom and *ξ*=1/2. Using equivalent operators we then find the first-order effective Hamiltonian 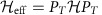 of equation (2). The only non-zero elements of the symmetric on-site tensor **Γ**_1_ are 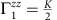, 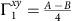 and 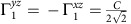, while those of Γ_2,***δ***_ are 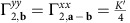, 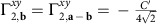, 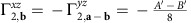 and 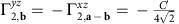. Finally, the intersite isotropic exchange interactions are *J*_**a**_=(*J*_2_+*J*_3_)/2, *J*_**a**−2**b**_=*J*_3_/4, *J*_**b**_=*J*_**a**−**b**_=*J*_2_/2+*J*′/4. We here employed the global coordinate system {**x**, **y**, **z**} corresponding to the Kitaev-like frame {**x**_*b*_, **y**_*b*_, **z**_*b*_} with *b*=B1 (Supplementary Figure 1). *J*′, *K*′, *A*′, *B*′ and *C*′ are effective coupling constants on the bonds B2 and B3, as also mentioned in the main text. We stress that the on-site quadrupolar term 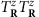 scales with *K*/2, while in the classical treatment of the original spin-1/2 model such a term would scale with *K*/4. We can trace this back to the value of *ξ*=1/2 found above, which in the classical treatment is *ξ*_clas_=1/4. This means that the quantum mechanical correlations strongly enhance the effect of the ‘on-site' anisotropy term *K*. The latter favours alignment along the *z* axis, against the effect of *K*′ which favours alignment within the *xy* plane. This point is further discussed in Supplementary Note 3 and 4, where we compare the classical treatment of the original spin-1/2 hexagonal model with the effective spin-1 triangular model.

## Additional information

**How to cite this article:** Nishimoto, S. *et al*. Strongly frustrated triangular spin lattice emerging from triplet dimer formation in honeycomb Li_2_IrO_3_. *Nat. Commun.* 7:10273 doi: 10.1038/ncomms10273 (2016).

## Supplementary Material

Supplementary Information Supplementary Figures 1-5, Supplementary Tables 1-3, Supplementary Notes 1-4 and Supplementary References

## Figures and Tables

**Table 1:** Magnetic spectra of two adjacent Ir^4+^ sites and effective exchange interaction parameters in Li_2_IrO_3_.

**Energies and effective couplings**	***b***=B1*	***b*****=B2/B3**†
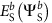	0.0	0.0
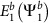	−17.1	1.3
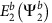	−24.8	−3.4
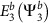	−21.6	−7.1
*J*_*b*_	−19.2	0.8
*K*_*b*_	−6.0	−11.6
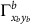	−1.1	4.2
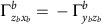	−4.8	−2.0

Relative energies of the four low-lying magnetic states and the associated effective exchange couplings (meV) for each of the two distinct types of (Ir_2_O_10_) units, B1 and B2/B3 (ref. 26), are shown. The energy of the singlet is taken as reference. Results of spin-orbit MRCI calculations.

^*^∡(Ir–O–Ir)=95.3°, *d*(Ir–Ir)=2.98 (× 2), *d*(Ir–O_1,2_)=2.01 Å.

^†^∡(Ir–O–Ir)=94.7°, *d*(Ir–Ir)=2.98 (× 4), *d*(Ir–O_1_)=2.08, *d*(Ir–O_2_)=1.97 Å. O_1_ and O_2_ are the two bridging O's.
