# Supplementary material for: Strongly frustrated triangular spin lattice emerging from triplet dimer formation in honeycomb Li2IrO3
Source: Nat Commun. 2016 Jan 18;7:10273. doi: 10.1038/ncomms10273 (PMC4735606; doi:10.1038/ncomms10273)
Supplement: Supplementary Information — Supplementary Figures 1-5, Supplementary Tables 1-3, Supplementary Notes 1-4 and Supplementary References [file ncomms10273-s1.pdf]

Supplementary Information

Supplementary Figures:

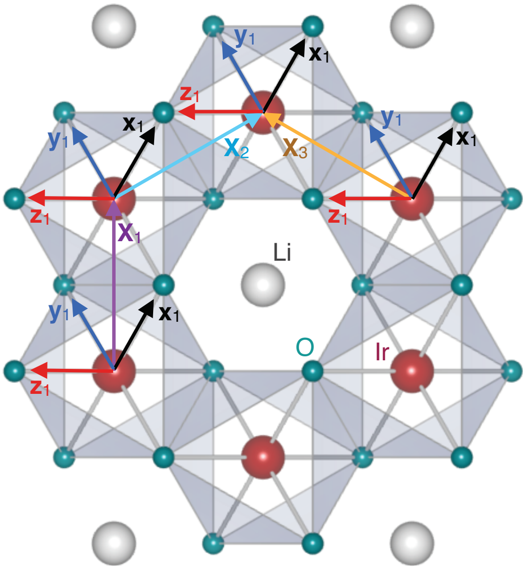

Supplementary Figure 1: **Reference frames used to express the the effective spin Hamiltonian.** The two different local reference frames $\{\mathbf{X}_b, \mathbf{Y}_b, \mathbf{Z}_b\}$ and $\{\mathbf{x}_b, \mathbf{y}_b, \mathbf{z}_b\}$ introduced for each of the three different types of nearest-neighbor Ir-Ir links are shown.

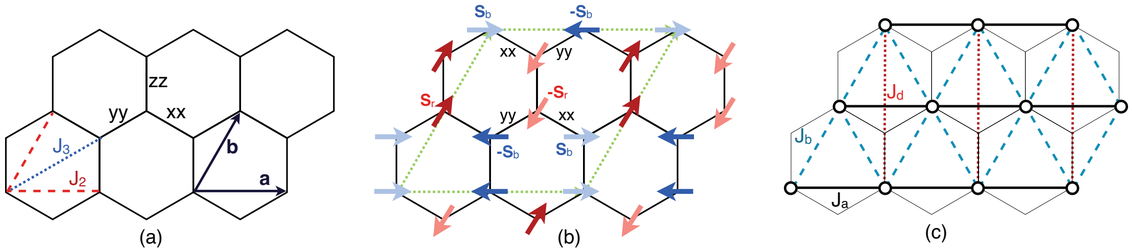

Supplementary Figure 2: **Exchange couplings in the honeycomb and the effective triangular lattices.** (a) Honeycomb lattice model of Li213. There are three types of NN Ir-Ir links, xx, yy, and zz, with $K^{(zz)} \equiv K$, $K^{(xx)} = K^{(yy)} \equiv K'$ and similarly $J^{(xx)} \equiv J$, $J^{(yy)} = J^{(zz)} \equiv J'$ (see also Supplementary Note II). Longer-range J_2 and J_3 couplings are also indicated. The vectors $\mathbf{a} = \mathbf{x}$ and $\mathbf{b} = (\mathbf{x} + \sqrt{3}\mathbf{y})/2$ are primitive translations of the lattice. (b) Classical limit of large and negative J and positive J_3. (c) Spin-1 model with effective couplings $J_a = (J_2 + J_3)/2$ along $\mathbf{a}$, $J_b = J_2/2 + J'/4$ along $\mathbf{b}$ and $\mathbf{c} = \mathbf{a} - \mathbf{b}$ plus the longer-range effective coupling $J_d = J_3/4$ along the vector $\mathbf{d} = \mathbf{a} - 2\mathbf{b}$.

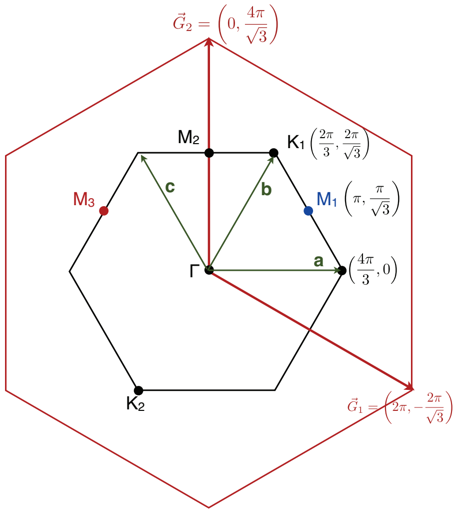

Supplementary Figure 3: **Brillouin zones of the honeycomb and triangular lattices.** The first (inner black hexagon) and second (outer red hexagon) Brillouin zones are illustrated.

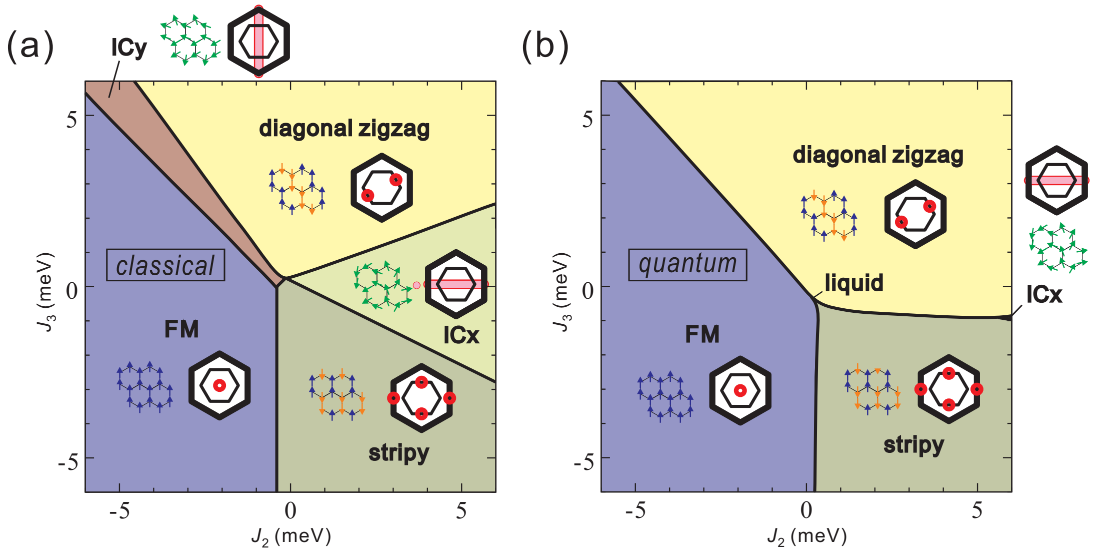

Supplementary Figure 4: **Magnetic phase diagrams in the limit** $C = D = C' = D' = 0$**.** (a) Classical phase diagram of the effective spin-1 model. (b) Quantum mechanical phase diagram of the original spin-1/2 model, as found by exact diagonalizations on the 24-site cluster (see details in main text).

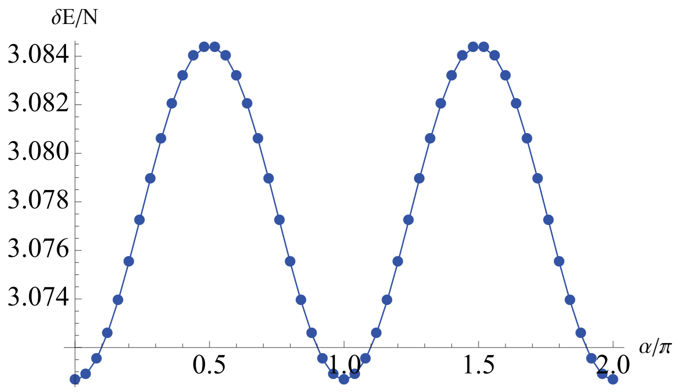

Supplementary Figure 5: **Energy correction.** Quadratic energy correction δE per site (in units of $|J|=1$) as a function of the relative angle α between the blue and red sublattices.

Supplementary Tables:

Supplementary Table 1: **Exchange coupling parameters as obtained at the MRCI level of theory.** Units of meV are used.

Bond type b	$J_b^{(0)}$	A_b	B_b	C_b	J_b=$J_b^{(0)}$+$\frac{A_b+B_b}{2}$	$K_b = -\frac{3}{2}(A_b + B_b)$	$D_b = \frac{A-B}{2}$
B1($b = 1$)	–21.17	0.87	3.11	6.84	–19.2	–6	–1.1
B2($b = 2$)	–3.07	8.07	–0.34	2.91	0.8	–11.59	+4.2
B3($b = 3$)	–3.07	8.07	–0.34	2.91	0.8	–11.59	+4.2

Supplementary Table 2: **Exchange coupling parameters as obtained at the CASSCF level of theory.** Units of meV are used.

Bond type b	$J_b^{(0)}$	A_b	B_b	C_b	J_b=$J_b^{(0)}$+$\frac{A_b+B_b}{2}$	$K_b = -\frac{3}{2}(A_b + B_b)$	$D_b = \frac{A-B}{2}$
B1($b = 1$)	–23.17	1.47	0.78	5.18	–22.0	–3.4	+0.3
B2($b = 2$)	–3.07	5.67	–0.69	1.95	–0.6	–7.5	+3.2
B3($b = 3$)	–3.07	5.67	–0.69	1.95	–0.6	–7.5	+3.2

Supplementary Table 3: **Exchange couplings calulated for different Ir-O-Ir bond angles.** Idealized crystal structures are used for both Na_2IrO_3 and Li_2IrO_3, see main text.

Na_2IrO_3						Li_2IrO_3					
$\measuredangle$(Ir-O-Ir)°	J	K	Γ_{xy}	Γ_{zx}	Γ_{yz}	$\measuredangle$(Ir-O-Ir)°	J	K	Γ_{xy}	Γ_{zx}	Γ_{yz}
90.0	0.32	–0.43	2.54	–1.33	1.33	90.0	0.40	–1.60	5.40	–2.82	2.82
91.0	–0.70	–2.19	2.60	–1.33	1.33	91.4	–3.27	–3.19	5.13	–2.75	2.75
92.5	–2.30	–4.40	2.60	–1.51	1.51	93.0	–5.23	–6.81	4.87	–3.10	3.10
94.0	–2.81	–7.18	2.39	–1.77	1.77	94.5	–5.32	–10.74	4.38	–3.45	3.45
95.5	–2.54	–9.87	2.16	–2.08	2.08	96.0	–3.70	–15.10	3.60	–4.01	4.01
97.0	–1.31	–12.97	1.79	–2.53	2.53	97.5	–1.00	–19.71	2.70	–4.66	4.66
100	2.59	–19.88	0.49	–3.93	3.93	99.0	2.41	–24.64	1.61	–5.56	5.56

Supplementary Notes:

Supplementary Note 1: Quantum chemistry calculations

In the *ab initio* quantum chemistry calculations we used energy-consistent relativistic pseudopotentials with quadruple-zeta basis sets for the valence shells of the two reference Ir ions [1], all-electron quintuple-zeta basis sets for the bridging ligands [2] and triple-zeta basis functions for the other O's of the two reference octahedra [2]. We also applied polarization functions for the central Ir ions and the two bridging ligands, two Ir f and four O d functions [1, 2]. For the NN Ir^{4+} and Li^{+} ions we employed total-ion effective potentials supplemented with $[2s2p2d]$ and $[1s]$ valence basis functions, respectively (Stoll 2013) [3]. Identical basis sets were used for Ir and O in the calculations for the idealized structures of Na213 (Fig. 2 in main text), with the same type of Na pseudopotentials and basis functions as for the Li ions in Li213.

As mentioned in the main text, the multireference configuration-interaction (MRCI) treatment includes single and double excitations from the Ir t_{2g} shells and the $2p$ orbitals of the bridging ligands. A similar strategy of explicitly dealing only with selected groups of localized ligand orbitals was earlier adopted in quantum chemistry studies on both $3d$ [4–7] and $5d$ [8–12] compounds, with results in good agreement with the experiment [4–10]. To separate the Ir $5d$ and O $2p$ valence orbitals into different groups, we used the Pipek-Mezey [13] orbital localization module available in MOLPRO [14].

The effective magnetic Hamiltonian for two adjacent Ir sites is most conveniently written in the local reference frame $\{\mathbf{X}_b,\mathbf{Y}_b,\mathbf{Z}_b\}$ with the $\mathbf{X}_b$ axis along the Ir-Ir bond and $\mathbf{Z}_b$ perpendicular to the Ir_2O_2 plaquette (see Methods in the main text). For C_{2h} point-group symmetry, it reads

$$\mathcal{H}_{\langle ij\rangle} = \tilde{\mathbf{S}}_i \cdot \begin{pmatrix} J^{(0)}+A & 0 & 0 \\ 0 & J^{(0)}+B & C \\ 0 & C & J^{(0)}-(A+B) \end{pmatrix} \cdot \tilde{\mathbf{S}}_j \,. \quad (1)$$

For simplicity, we omitted the bond index b. A straightforward diagonalization of $\mathcal{H}_{\langle ij\rangle}$ yields the following eigenvalues and eigenfunctions:

$$\begin{aligned} E_{\rm S} &= -\frac{3J^{(0)}}{4}\,, & \Phi_{\rm S} &= \frac{\uparrow\downarrow-\downarrow\uparrow}{\sqrt{2}}, \\ E_1 &= \frac{J^{(0)}+A+\sqrt{(A+2B)^2+4C^2}}{4}\,, & \Phi_1 &= \frac{\uparrow\downarrow+\downarrow\uparrow}{\sqrt{2}}, \\ E_2 &= \frac{J^{(0)}+A-\sqrt{(A+2B)^2+4C^2}}{4}\,, & \Phi_2 &= \frac{\uparrow\uparrow+\downarrow\downarrow}{\sqrt{2}}, \\ E_3 &= \frac{J^{(0)}-2A}{4}\,, & \Phi_3 &= \frac{\uparrow\uparrow-\downarrow\downarrow}{\sqrt{2}}. \end{aligned} \quad (2)$$

Here $\Phi_{\rm S}$ is the total spin singlet and Φ_{1-3} are combinations of the three triplet components. The latter are degenerate in the plain Heisenberg model.

The above diagonalization procedure is equivalent with a rotation of the coordinate system $\{\mathbf{X},\mathbf{Y},\mathbf{Z}\}$ around $\mathbf{X}$ by an angle α to a new frame $\{\mathbf{X}',\mathbf{Y}',\mathbf{Z}'\}$ in which the symmetric anisotropic exchange matrix is diagonal [15]. $\{\mathbf{X}',\mathbf{Y}',\mathbf{Z}'\}$ are also referred to as principal axes and the angle α is given by

$$\tan(2\alpha) = \frac{2C}{A+2B}\,. \quad (3)$$

In C_{2h} symmetry, the $\Phi_{\rm S}$, Φ_1, Φ_2 and Φ_3 spin-orbit wave functions transform according to the A_g, B_u, B_u and A_u irreducible representations, respectively. Since states Φ_1 and Φ_2 belong to the same irreducible representation B_u, they are in general admixed, i.e., in the reference frame $\{\mathbf{X},\mathbf{Y},\mathbf{Z}\}$ the corresponding eigenfunctions should be written as

$$\begin{aligned} \Psi_1 &= \Phi_1\cos\alpha + i\Phi_2\sin\alpha\,, \\ \Psi_2 &= i\Phi_1\sin\alpha + \Phi_2\cos\alpha\,. \end{aligned} \quad (4)$$

The mixing parameter $\zeta = \sin\alpha$ is given by

$$i\zeta = \langle\Phi_2|\Psi_1\rangle = \langle\Phi_1|\Psi_2\rangle\,. \quad (5)$$

Since the quantum chemistry calculations were actually performed in C_1 symmetry, to determine the nature of each of the lowest four spin-orbit states we explicitly computed the dipole and quadrupole transition matrix elements within that manifold. Standard selection rules and the nonzero dipole and quadrupole matrix elements in the quantum chemistry outputs then clearly indicate which state is which. We also carried out the transformation of the spin-orbit wave functions from the usual $\{L_1,M_{L_1},L_2,M_{L_2},S,M_S\}$ basis in standard quantum chemistry programs to the $\{\tilde{S}_1,\tilde{S}_2,\tilde{M}_{S_1},\tilde{M}_{S_2}\}$ basis. This allows the study of Φ_1–Φ_2 mixing due to the offdiagonal Γ_{yz} and Γ_{zx} couplings.

Supplementary Note 2: Lattice spin model, conventions and *ab initio* effective couplings

For each type of Ir-Ir link, we used two different local axes frames, namely $\{\mathbf{x}_b, \mathbf{y}_b, \mathbf{z}_b\}$ and $\{\mathbf{X}_b, \mathbf{Y}_b, \mathbf{Z}_b\} = \{\frac{\mathbf{x}_b+\mathbf{y}_b}{\sqrt{2}}, \frac{-\mathbf{x}_b+\mathbf{y}_b}{\sqrt{2}}, \mathbf{z}_b\}$, where $b \in \{B1 \equiv 1, B2 \equiv 2, B3 \equiv 3\}$. Let us choose our global frame to be $\{\mathbf{x}, \mathbf{y}, \mathbf{z}\} = \{\mathbf{x}_1, \mathbf{y}_1, \mathbf{z}_1\}$. The relation between the local bond axes $\{\mathbf{X}_b, \mathbf{Y}_b, \mathbf{Z}_b\}$ and the global coordinate system is the following (see Supplementary Figure 1):

$$\{\mathbf{X}_1, \mathbf{Y}_1, \mathbf{Z}_1\} = \{\frac{\mathbf{x}+\mathbf{y}}{\sqrt{2}}, \frac{-\mathbf{x}+\mathbf{y}}{\sqrt{2}}, \mathbf{z}\}, \;\; \{\mathbf{X}_2, \mathbf{Y}_2, \mathbf{Z}_2\} = \{\frac{\mathbf{x}-\mathbf{z}}{\sqrt{2}}, -\frac{\mathbf{x}+\mathbf{z}}{\sqrt{2}}, \mathbf{y}\}, \;\; \{\mathbf{X}_3, \mathbf{Y}_3, \mathbf{Z}_3\} = \{\frac{\mathbf{y}+\mathbf{z}}{\sqrt{2}}, \frac{-\mathbf{y}+\mathbf{z}}{\sqrt{2}}, \mathbf{x}\}. \quad (6)$$

We note that the system is invariant under two-fold rotations around the vector $\mathbf{X}_1$. This is why we must choose $\mathbf{X}_3$ to be the rotated version of $\mathbf{X}_2$, i.e., $\mathbf{C}_2 \cdot \mathbf{X}_2 = \mathbf{X}_3$. If we adopt the opposite direction for $\mathbf{X}_3$ then we must change the sign of the coupling C.

Let us now write down the NN terms of the Hamiltonian for the three types of bonds $b = 1, 2, 3$. In the reference frame $\{\mathbf{X}_b, \mathbf{Y}_b, \mathbf{Z}_b\}$,

$$\begin{aligned} \mathcal{H}_{\langle ij\rangle \in b} &= J_b^{(0)} \mathbf{S}_i \cdot \mathbf{S}_j + A_b S_i^{X_b} S_j^{X_b} + B_b S_i^{Y_b} S_j^{Y_b} - (A_b + B_b) S_i^{Z_b} S_j^{Z_b} + C_b (S_i^{Y_b} S_j^{Z_b} + S_i^{Z_b} S_j^{Y_b}) \\ &= J_b \mathbf{S}_i \cdot \mathbf{S}_j + K_b S_i^{Z_b} S_j^{Z_b} + D_b (S_i^{X_b} S_j^{X_b} - S_i^{Y_b} S_j^{Y_b}) + C_b (S_i^{Y_b} S_j^{Z_b} + S_i^{Z_b} S_j^{Y_b}) \,, \end{aligned} \quad (7)$$

where $J_b = J_b^{(0)} + \frac{1}{2}(A_b + B_b)$, $K_b = -\frac{3}{2}(A_b + B_b)$ and $D_b = \frac{1}{2}(A_b - B_b)$. For simplicity, we replace $J_{b=1}$ by J, $J_{b=2,3}$ by J' and similarly for the remaining parameters. Supplementary Equations (7) and (6) then yield

$$\mathcal{H}_{\langle ij\rangle \in b=1} = J \mathbf{S}_i \cdot \mathbf{S}_j + K S_i^z S_j^z + D(S_i^x S_j^y + S_i^y S_j^x) + \frac{C}{\sqrt{2}} (S_i^y S_j^z + S_i^z S_j^y - S_i^x S_j^z - S_i^z S_j^x) \,,$$

$$\mathcal{H}_{\langle ij\rangle \in b=2} = J' \mathbf{S}_i \cdot \mathbf{S}_j + K' S_i^y S_j^y + D'(-S_i^x S_j^z - S_i^z S_j^x) + \frac{C'}{\sqrt{2}} (-S_i^x S_j^y - S_i^y S_j^x - S_i^z S_j^y - S_i^y S_j^z) \,,$$

$$\mathcal{H}_{\langle ij\rangle \in b=3} = J' \mathbf{S}_i \cdot \mathbf{S}_j + K' S_i^x S_j^x + D'(S_i^y S_j^z + S_i^z S_j^y) + \frac{C'}{\sqrt{2}} (-S_i^x S_j^y - S_i^y S_j^x + S_i^x S_j^z + S_i^z S_j^x) \,,$$

where $D = \frac{A-B}{2}$ and $D' = \frac{A'-B'}{2}$. With the conventions introduced in Supplementary Figure 1, the spin Hamiltonian now reads:

$$\begin{aligned} \mathcal{H} = \sum_{\mathbf{R}} & J \, \mathbf{S}_{\mathbf{R},1} \cdot \mathbf{S}_{\mathbf{R},2} + K \, S^z_{\mathbf{R},1} S^z_{\mathbf{R},2} + D (S^x_{\mathbf{R},1} S^y_{\mathbf{R},2} + S^y_{\mathbf{R},1} S^x_{\mathbf{R},2}) \\ & + \frac{C}{\sqrt{2}} (S^y_{\mathbf{R},1} S^z_{\mathbf{R},2} + S^z_{\mathbf{R},1} S^y_{\mathbf{R},2} - S^x_{\mathbf{R},1} S^z_{\mathbf{R},2} - S^z_{\mathbf{R},1} S^x_{\mathbf{R},2}) \\ & + J' \, \mathbf{S}_{\mathbf{R},1} \cdot \mathbf{S}_{\mathbf{R}-\mathbf{b},2} + K' \, S^y_{\mathbf{R},1} S^y_{\mathbf{R}-\mathbf{b},2} + D' (-S^x_{\mathbf{R},1} S^z_{\mathbf{R}-\mathbf{b},2} - S^z_{\mathbf{R},1} S^x_{\mathbf{R}-\mathbf{b},2}) \\ & + \frac{C'}{\sqrt{2}} (-S^x_{\mathbf{R},1} S^y_{\mathbf{R}-\mathbf{b},2} - S^y_{\mathbf{R},1} S^x_{\mathbf{R}-\mathbf{b},2} - S^z_{\mathbf{R},1} S^y_{\mathbf{R}-\mathbf{b},2} - S^y_{\mathbf{R},1} S^z_{\mathbf{R}-\mathbf{b},2}) \\ & + J' \, \mathbf{S}_{\mathbf{R},1} \cdot \mathbf{S}_{\mathbf{R}+\mathbf{a}-\mathbf{b},2} + K' \, S^x_{\mathbf{R},1} S^x_{\mathbf{R}+\mathbf{a}-\mathbf{b},2} + D' (S^y_{\mathbf{R},1} S^z_{\mathbf{R}+\mathbf{a}-\mathbf{b},2} + S^z_{\mathbf{R},1} S^y_{\mathbf{R}+\mathbf{a}-\mathbf{b},2}) \\ & + \frac{C'}{\sqrt{2}} (-S^x_{\mathbf{R},1} S^y_{\mathbf{R}+\mathbf{a}-\mathbf{b},2} - S^y_{\mathbf{R},1} S^x_{\mathbf{R}+\mathbf{a}-\mathbf{b},2} + S^x_{\mathbf{R},1} S^z_{\mathbf{R}+\mathbf{a}-\mathbf{b},2} + S^z_{\mathbf{R},1} S^x_{\mathbf{R}+\mathbf{a}-\mathbf{b},2}) \\ & + J_2 \, \mathbf{S}_{\mathbf{R},1} \cdot (\mathbf{S}_{\mathbf{R}+\mathbf{a},1} + \mathbf{S}_{\mathbf{R}+\mathbf{b},1} + \mathbf{S}_{\mathbf{R}+\mathbf{a}-\mathbf{b},1}) + J_2 \, \mathbf{S}_{\mathbf{R},2} \cdot (\mathbf{S}_{\mathbf{R}+\mathbf{a},2} + \mathbf{S}_{\mathbf{R}+\mathbf{b},2} + \mathbf{S}_{\mathbf{R}+\mathbf{a}-\mathbf{b},2}) \\ & + J_3 \, \mathbf{S}_{\mathbf{R},1} \cdot (\mathbf{S}_{\mathbf{R}+\mathbf{a},2} + \mathbf{S}_{\mathbf{R}-\mathbf{a},2} + \mathbf{S}_{\mathbf{R}+\mathbf{a}-2\mathbf{b},2}) \,, \end{aligned} \quad (8)$$

where $\mathbf{R} = n\mathbf{a} + m\mathbf{b}$ labels the unit cells and $j = 1-2$ labels the sublattice index.

The numerical values of the above coupling parameters are provided in Supplementary Tables 1 and 2.

Supplementary Note 3: Effective spin-1 description in momentum space

As mentioned in the main text, the effective spin-1 model takes the following form in momentum space:

$$\mathcal{H}_{\text{eff}} = \sum_{\alpha,\beta,\mathbf{k}} T^{\alpha}_{\mathbf{k}} \, \Lambda_{\alpha\beta}(\mathbf{k}) \, T^{\beta}_{-\mathbf{k}} \,, \tag{9}$$

where $\mathbf{T}_{\mathbf{R}} = \frac{1}{\sqrt{N}} \sum_{\mathbf{k}} e^{-i\mathbf{k}\cdot\mathbf{R}} \mathbf{T}_{\mathbf{k}}$, N is the number of *B1* bonds on the lattice, the matrix $\mathbf{\Lambda}$ is given by

$$\mathbf{\Lambda}(\mathbf{k}) = \begin{pmatrix} f(\mathbf{k}) + \frac{K'}{4}\cos(\mathbf{k}\cdot\mathbf{c}) & \frac{D}{2} - \frac{C'}{4\sqrt{2}}[\cos(\mathbf{k}\cdot\mathbf{b}) + \cos(\mathbf{k}\cdot\mathbf{c})] & -\frac{C}{2\sqrt{2}} + \frac{C'}{4\sqrt{2}}\cos(\mathbf{k}\cdot\mathbf{c}) - \frac{D'}{4}\cos(\mathbf{k}\cdot\mathbf{b}) \\ \frac{D}{2} - \frac{C'}{4\sqrt{2}}[\cos(\mathbf{k}\cdot\mathbf{b}) + \cos(\mathbf{k}\cdot\mathbf{c})] & f(\mathbf{k}) + \frac{K'}{4}\cos(\mathbf{k}\cdot\mathbf{b}) & \frac{C}{2\sqrt{2}} - \frac{C'}{4\sqrt{2}}\cos(\mathbf{k}\cdot\mathbf{b}) + \frac{D'}{4}\cos(\mathbf{k}\cdot\mathbf{c}) \\ -\frac{C}{2\sqrt{2}} + \frac{C'}{4\sqrt{2}}\cos(\mathbf{k}\cdot\mathbf{c}) - \frac{D'}{4}\cos(\mathbf{k}\cdot\mathbf{b}) & \frac{C}{2\sqrt{2}} - \frac{C'}{4\sqrt{2}}\cos(\mathbf{k}\cdot\mathbf{b}) + \frac{D'}{4}\cos(\mathbf{k}\cdot\mathbf{c}) & f(\mathbf{k}) + \frac{K}{2} \end{pmatrix},$$

$$f(\mathbf{k}) = \frac{J_2 + J_3}{2}\cos(\mathbf{k}\cdot\mathbf{a}) + \frac{J_3}{4}\cos(\mathbf{k}\cdot\mathbf{d}) + \left(\frac{J_2}{2} + \frac{J'}{4}\right)\left[\cos(\mathbf{k}\cdot\mathbf{b}) + \cos(\mathbf{k}\cdot\mathbf{c})\right]$$

and $\mathbf{c} = \mathbf{a} - \mathbf{b}$, $\mathbf{d} = \mathbf{a} - 2\mathbf{b}$.

The classical phase diagram of $\mathcal{H}_{\text{eff}}$ can be investigated by a numerical minimization of the eigenvalues of $\mathbf{\Lambda}(\mathbf{k})$ over the entire Brillouin zone (BZ) of the triangular lattice (shown in Supplementary Figure 3), which is essentially the Luttinger-Tisza method [16] for a Hamiltonian with anisotropic terms in spin space. In contrast to isotropic Hamiltonians, here not all directions in spin space are equivalent. This is an important aspect reflected in the form of the eigenvectors of $\mathbf{\Lambda}$.

When the minimum sits at a commensurate wavevector $\mathbf{Q}$ — as in the ferromagnetic (FM), diagonal zigzag and stripy phases — the ground state is given by the corresponding commensurate modulation (see section 1 below) and the actual directions in spin space are set by the eigenvector corresponding to the minimum eigenvalue. If $\mathbf{Q}$ is an incommensurate point and the minimum eigenvalue at $\mathbf{Q}$ is at least two-fold degenerate then the ground state is given by a spiral configuration in the plane formed by the corresponding eigenvectors. The situation is more complex when $\mathbf{Q}$ is an incommensurate point and the minimum eigenvalue is non-degenerate, as in the regions ICx and ICy. In this case we cannot write down a spiral solution that satisfies the spin length constraint $|\mathbf{T}_{\mathbf{R}}| = 1$ for all $\mathbf{R}$ and the actual nature of the ground state goes beyond the standard helical configuration (see also main text).

The classical phase diagram for the case of $C = D = C' = D' = 0$ is provided in Supplementary Figure 4(a), while the one for finite C, D, C' and D' is shown in Figure 3 of the main text. In the same figure we show the quantum phase diagram of the original spin-1/2 model, as obtained from exact diagonalization (ED) calculations on the 24-site cluster. As for the case of finite C, D, C' and D', we do not find any traces of the incommensurate phase ICy in the ED calculations. This is either a finite-size effect or due to the fact that the classical ICy region is very narrow, meaning that the competing nearby FM and zigzag phases are very close in energy and are more likely to be favored by quantum mechanical fluctuations. The surprising result is that the quantum ICx region is extremely narrow for small J_2 and J_3, drastically different from what we found for finite C, D, C' and D'. Since the classical minimization does not show such a drastic dependence of the ICx region on C, D, C' and D', we can attribute this to finite-size effects in the ED calculations.

In the following two subsections we shall focus on the zigzag phase to demonstrate what happens for the simplest, commensurate cases.

Zigzag phase – limit of $C = C' = D = D' = 0$

In this limit, $\mathbf{\Lambda}(\mathbf{k})$ becomes diagonal:

$$\mathbf{\Lambda}(\mathbf{k}) = \begin{pmatrix} f(\mathbf{k}) + \frac{K'}{4}\cos(\mathbf{k}\cdot\mathbf{c}) & 0 & 0 \\ 0 & f(\mathbf{k}) + \frac{K'}{4}\cos(\mathbf{k}\cdot\mathbf{b}) & 0 \\ 0 & 0 & f(\mathbf{k}) + \frac{K}{2} \end{pmatrix}. \tag{10}$$

Now, the minimum of $f(\mathbf{k})$ is at the two M points $\mathbf{M}_1 = (\pi, \frac{\pi}{\sqrt{3}})$ and $\mathbf{M}_3 = (-\pi, \frac{\pi}{\sqrt{3}})$ (see Supplementary Figure 3), with an eigenvalue $\lambda_0 = -\frac{J_2+J_3}{2} - \frac{J_3}{4}$. With $\mathbf{M}_1 \perp \mathbf{c}$ and $\mathbf{M}_3 \perp \mathbf{b}$, the minimum of $L_x(\mathbf{k}) \equiv f(\mathbf{k}) + \frac{K'}{4}\cos(\mathbf{k}\cdot\mathbf{c})$ is at $\mathbf{M}_1$, with an eigenvalue $\lambda_x = \lambda_0 - \frac{|K'|}{4}$. The minimum of $L_y(\mathbf{k}) \equiv f(\mathbf{k}) + \frac{K'}{4}\cos(\mathbf{k}\cdot\mathbf{b})$ is at $\mathbf{M}_3$, with an eigenvalue $\lambda_y = \lambda_x$. Finally, $L_z(\mathbf{k}) \equiv f(\mathbf{k}) + \frac{K}{2}$ is minimum at both $\mathbf{M}_1$ and $\mathbf{M}_3$, with the eigenvalue $\lambda_z = \lambda_0 - \frac{|K|}{2}$. Since $|K|/2 > |K'|/4$, the lowest

eigenvalue is λ_z. This means that spins will prefer to point along the z axis and not within the xy-plane. Still, there are two M points available and we therefore have two possible solutions:

$$\mathbf{T}(n\mathbf{a}+m\mathbf{b}) = e^{i\mathbf{M}_1\cdot\mathbf{r}}\mathbf{e}_z = (-1)^{n+m}\mathbf{e}_z\,, \quad \mathbf{T}(n\mathbf{a}+m\mathbf{b}) = e^{i\mathbf{M}_3\cdot\mathbf{r}}\mathbf{e}_z = (-1)^{n}\mathbf{e}_z\,, \tag{11}$$

where $\mathbf{R} = n\mathbf{a}+m\mathbf{b}$ and n, m are integers.

Remark 1: The direction in spin space basically boils down to the competition between the two anisotropic Kitaev terms $|K|/2$ and $|K'|/4$. The difference in the two prefactors by a factor of 2 goes back to the value of $\xi = 1/2$ in the *equivalent operator* for the onsite quadrupolar terms. This difference is a quantum mechanical effect since, as we show below, in the classical treatment of the original spin-1/2 model, the direction in spin space is actually fixed by the competition between $|K|/4$ and $|K'|/4$.

Remark 2: If the couplings K and K' were such that $|K|/2 < |K'|/4$, $\lambda_{x,y}$ would be the lowest eigenvalues. Then the two solutions from the two M points would give

$$\mathbf{T}(n\mathbf{a}+m\mathbf{b}) = e^{i\mathbf{M}_1\cdot\mathbf{r}}\mathbf{e}_x = (-1)^{n+m}\mathbf{e}_x\,, \quad \mathbf{T}(n\mathbf{a}+m\mathbf{b}) = e^{i\mathbf{M}_1\cdot\mathbf{r}}\mathbf{e}_y = (-1)^{n}\mathbf{e}_y\,. \tag{12}$$

In this case we can in fact combine the two solutions to get a one-parameter family of degenerate states

$$\mathbf{T}(n\mathbf{a}+m\mathbf{b}) = \cos\alpha\,(-1)^{n+m}\mathbf{e}_x + \sin\alpha\,(-1)^{n}\mathbf{e}_y \tag{13}$$

that contains coplanar as well as collinear states. The quantum order-by-disorder effect would most likely select the collinear states $\alpha = 0$ or π, as they do in the original spin-1/2 model, see below.

Zigzag phase – finite C, D, C' and D'

Diagonalizing the matrix $\mathbf{\Lambda}(\mathbf{k})$ we find that the minimum eigenvalue occurs again at the points $\mathbf{M}_1$ and $\mathbf{M}_3$. The difference with the previous case is that now the principal eigenvectors differ from the $(1,0,0)$, $(0,0,1)$ or $(0,0,1)$ directions. For example, for $J_2 = J_3 = 3$ meV, we obtain the following eigensolutions:

$$\mathbf{M}_1: \quad \lambda_1 = -8.87983, \quad \mathbf{v}_1 = (0.232318, -0.41947, 0.877538)\,; \tag{14}$$

$$\mathbf{M}_3: \quad \lambda_3 = -8.87983, \quad \mathbf{v}_3 = (0.41947, -0.232318, 0.877538)\,. \tag{15}$$

This means that the spatial arrangement of the spins is that of the *diagonal stripy* phase but the overall direction in spin space is now along $\mathbf{v}_1$ or $\mathbf{v}_3$, which are tilted away from the z axis:

$$\mathbf{T}(n\mathbf{a}+m\mathbf{b}) = e^{i\mathbf{M}_1\cdot\mathbf{r}}\mathbf{v}_1 = (-1)^{n+m}\mathbf{v}_1, \quad \mathbf{T}(n\mathbf{a}+m\mathbf{b}) = e^{i\mathbf{M}_3\cdot\mathbf{r}}\mathbf{v}_3 = (-1)^{n}\mathbf{v}_3\,. \tag{16}$$

Supplementary Note 4: Back to the original spin-1/2 model: Classical limit of large $-J > 0$ and $J_3 > 0$

Here we discuss a semiclassical description of the *diagonal stripy* phase in the original spin-1/2 model. We begin by taking the limit $J' = K = K' = J_2 = D = D' = C = C' = 0$. The coupling J forces all pairs of spins on *B1* bonds to be aligned, see Supplementary Figure 2(b). Together with the large antiferromagnetic (AF) coupling J_3, these interactions give rise to two decoupled sublattices, denoted in Supplementary Figure 2(b) by blue arrows ($\pm\mathbf{S}_b$) and red arrows ($\pm\mathbf{S}_r$). Neither J nor J_3 can fix the relative orientation ϕ between blue and red spins. So, let us see what the remaining couplings do at the classical level. For simplicity we replace $\mathbf{S}_b \to \mathbf{b}$ and $\mathbf{S}_r \to \mathbf{r}$.

- The contributions from the J' couplings cancel out.
- The contributions from the J_2 couplings between blue and red cancel out as well.
- The contributions from the J_2 couplings between blue with blue and red with red spins give an overall constant.
- The energy contribution from the K and K' couplings (where N stands for the number of *B1* bonds) is

$$E_K/N = \frac{1}{2}K\left[b_z^2 + r_z^2\right] + K'\left(-b_x r_x + b_y r_y\right)\,.$$

- The energy contribution from D and D' is

$$E_D/N = D\,(b_x b_y + r_x r_y) - D'\,(b_x r_z + b_z r_x + b_y r_z + b_z r_y)\ .$$

- The energy contribution from C and C' is

$$E_C/N = \frac{C}{\sqrt{2}}\,(b_y b_z - b_x b_z + r_y r_z - r_x r_z) - \frac{C'}{\sqrt{2}}\,(b_z r_y + b_y r_z + b_x r_z + b_z r_x)\ .$$

Altogether, in spherical coordinates:

$$\begin{aligned} E/N &= \frac{1}{2}K\left(\cos^2\theta_b + \cos^2\theta_r\right) - K'\sin\theta_b\sin\theta_r\cos(\phi_b+\phi_r) \\ &+ \frac{D}{2}\left(\sin^2\theta_b\sin(2\phi_b) + \sin^2\theta_r\sin(2\phi_r)\right) + C\left[\cos\theta_b\sin(\phi_b-\pi/4) + \cos\theta_r\sin(\phi_r-\pi/4)\right] \\ &- \left(\sqrt{2}D' + C'\right)\left[\cos\theta_b\cos(\phi_r-\pi/4) + \cos\theta_r\cos(\phi_b-\pi/4)\right]\ . \end{aligned} \tag{17}$$

The highly frustrated point $C = D = C' = D' = 0$**:** In this limit we have

$$E/N = \frac{1}{2}K\left(\cos^2\theta_b + \cos^2\theta_r\right) - K'\sin\theta_b\sin\theta_r\cos(\phi_b+\phi_r)\ . \tag{18}$$

Since the energy does not depend on the angle difference $\phi_b - \phi_r$, this limit features a ground state manifold with an accidental one-parameter family of degenerate states. If we require that the solutions have the property $\theta_b = \theta_r$ (confirmed by numerical minimization), then

$$E/N = K\cos^2\theta_b - K'\sin^2\theta_b\cos(\phi_b+\phi_r)\ . \tag{19}$$

The extremum value of the second term occurs for $|\cos(\phi_b+\phi_r)| = 1$. For $\cos(\phi_b+\phi_r) = 1$, we obtain

$$E_1/N = K\cos^2\theta_b - K'\sin^2\theta_b = -(|K| + |K'|)\cos^2\theta_b + |K'| \tag{20}$$

and the minimum is at $\theta_b = \theta_r = 0$ with $E_1 = -|K|$. On the other hand, for $\cos(\phi_b+\phi_r) = -1$, we find

$$E_2/N = K\cos^2\theta_b + K'\sin^2\theta_b = (|K'| - |K|)\cos^2\theta_b - |K'| \tag{21}$$

and, since $|K| < |K'|$, the minimum occurs for $\theta_b = \theta_r = \pi/2$ with $E_2 = -|K'|$. Comparing E_1 and E_2 we find that the minimum energy is E_2, i.e., when the spins lie within the xy-plane.

Quantum order-by-disorder — The above degeneracy of the classical ground state manifold is lifted by quantum fluctuations at the harmonic spin-wave level. This can be shown by performing a linear spin-wave expansion around the classical ground state manifold, which is parametrized by the angle α between the two sublattices. We follow the standard procedure and introduce two bosons $a_{\mathbf{R},j=1,2}$ per unit cell of the honeycomb lattice, followed by a Holstein-Primakoff expansion of the spin operators. The final form of the bosonic Hamiltonian reads

$$\begin{aligned} \mathcal{H} &\simeq S(S+1)\frac{E_{\mathsf{clas}}}{S^2} \\ &+ \frac{S}{2}\sum_{\mathbf{k}}\Big\{\Big[f_{1r}(\mathbf{k})(a_{\mathbf{k},1}a_{-\mathbf{k},1} + a_{\mathbf{k},2}a_{-\mathbf{k},2}) + f_2(\mathbf{k})a_{\mathbf{k},1}a_{-\mathbf{k},2} + f_2(-\mathbf{k})a_{-\mathbf{k},1}a_{\mathbf{k},2} + g_2(\mathbf{k})a_{\mathbf{k},1}a^{+}_{\mathbf{k},2} \\ &+ g_2(-\mathbf{k})a_{-\mathbf{k},1}a^{+}_{-\mathbf{k},2} + h.c.\Big] + g_1'(\mathbf{k})\Big[a^{+}_{\mathbf{k},1}a_{\mathbf{k},1} + a^{+}_{\mathbf{k},2}a_{\mathbf{k},2} + a_{-\mathbf{k},1}a^{+}_{-\mathbf{k},1} + a_{-\mathbf{k},2}a^{+}_{-\mathbf{k},2}\Big]\Big\} \\ &+ \mathcal{O}(\sqrt{S})\,, \end{aligned} \tag{22}$$

where $E_{\mathsf{clas}}/N = -S^2 B_{\mathsf{clas}}$, $B_{\mathsf{clas}} = -J + 2J_3 + 3J_3 - K'$, $f_{1r(\mathbf{k})} = \frac{f_1(\mathbf{k})+f_1(-\mathbf{k})}{2}$, $g_1'(\mathbf{k}) = g_1(k) + B_{\mathsf{clas}}$ and

$$f_1(\mathbf{k}) = 2J_2(e^{i\mathbf{k}\cdot\mathbf{a}} + \sin^2\frac{\alpha}{2}\,e^{i\mathbf{k}\cdot\mathbf{b}} + \cos^2\frac{\alpha}{2}\,e^{i\mathbf{k}\cdot\mathbf{c}})\,, \tag{23}$$

$$f_2(\mathbf{k}) = (J' + \frac{K'}{2})(\sin^2\frac{\alpha}{2}\,e^{-i\mathbf{k}\cdot\mathbf{b}} + \cos^2\frac{\alpha}{2}\,e^{i\mathbf{k}\cdot\mathbf{c}}) + J_3(e^{i\mathbf{k}\cdot\mathbf{a}} + e^{-i\mathbf{k}\cdot\mathbf{a}} + e^{i\mathbf{k}\cdot(\mathbf{a}-2\mathbf{b})}) + \frac{K}{2}\,, \tag{24}$$

$$g_1(\mathbf{k}) = J_2(\sin^2\frac{\alpha}{2}\,e^{i\mathbf{k}\cdot\mathbf{c}} + \cos^2\frac{\alpha}{2}\,e^{i\mathbf{k}\cdot\mathbf{b}}) + h.c. = 2J_2[\sin^2\frac{\alpha}{2}\,\cos(\mathbf{k}\cdot\mathbf{c}) + \cos^2\frac{\alpha}{2}\,\cos(\mathbf{k}\cdot\mathbf{b})]\,, \tag{25}$$

$$g_2(\mathbf{k}) = J + J'(\sin^2\frac{\alpha}{2}e^{i\mathbf{k}\cdot\mathbf{c}} + \cos^2\frac{\alpha}{2}e^{-i\mathbf{k}\cdot\mathbf{b}}) + \frac{K}{2} - \frac{K'}{2}(\cos^2\frac{\alpha}{2}\,e^{i\mathbf{k}\cdot\mathbf{c}} + \sin^2\frac{\alpha}{2}\,e^{-i\mathbf{k}\cdot\mathbf{b}})\ . \tag{26}$$

We may now group all terms in matrix form as

$$\mathcal{H} = S(S+1)\frac{E_{\mathsf{clas}}}{S^2} + \frac{S}{2}\sum_{\mathbf{k}} \mathbf{A}^+_{\mathbf{k}} \cdot \mathbf{M}_{\mathbf{k}} \cdot \mathbf{A}_{\mathbf{k}} + \mathcal{O}(\sqrt{S})\,, \tag{27}$$

with

$$\mathbf{A}_{\mathbf{k}} = \begin{pmatrix} a_{\mathbf{k},1} \\ a_{\mathbf{k},2} \\ a^+_{-\mathbf{k},1} \\ a^+_{-\mathbf{k},2} \end{pmatrix}, \quad \mathbf{M}_{\mathbf{k}} = \begin{pmatrix} g_1'(\mathbf{k}) & g_2(\mathbf{k}) & f_{1r}(\mathbf{k}) & f_2(\mathbf{k}) \\ g_2(-\mathbf{k}) & g_1'(\mathbf{k}) & f_2(-\mathbf{k}) & f_{1r}(\mathbf{k}) \\ f_{1r}(\mathbf{k}) & f_2(\mathbf{k}) & g_1'(\mathbf{k}) & g_2(\mathbf{k}) \\ f_2(-\mathbf{k}) & f_{1r}(\mathbf{k}) & g_2(-\mathbf{k}) & g_1'(\mathbf{k}) \end{pmatrix}, \quad \mathbf{A}^+_{\mathbf{k}} = \begin{pmatrix} a^+_{\mathbf{k},1}, & a^+_{\mathbf{k},2}, & a_{-\mathbf{k},1}, & a_{-\mathbf{k},2} \end{pmatrix}. \tag{28}$$

We next define the commutator matrix

$$\mathbf{g} = \mathbf{A}_{\mathbf{k}} \cdot \mathbf{A}^\dagger_{\mathbf{k}} - \left((\mathbf{A}^\dagger_{\mathbf{k}})^T \cdot \mathbf{A}^T_{\mathbf{k}}\right)^T = \left(\begin{array}{c|c} \mathbb{1} & 0 \\ \hline 0 & -\mathbb{1} \end{array}\right) \tag{29}$$

and search for a transformation $\mathbf{A}_{\mathbf{k}} = \mathbf{S}_{\mathbf{k}} \cdot \tilde{\mathbf{A}}_{\mathbf{k}}$ that conserves the bosonic commutation relations (i.e., $\tilde{\mathbf{g}} = \mathbf{g}$) and at the same time diagonalizes the Hamiltonian. Specifically, $\tilde{\mathbf{A}}^\dagger_{\mathbf{k}} \cdot (\mathbf{S}^\dagger_{\mathbf{k}} \mathbf{M}_{\mathbf{k}} \mathbf{S}_{\mathbf{k}}) \cdot \tilde{\mathbf{A}}_{\mathbf{k}} = \tilde{\mathbf{A}}^\dagger_{\mathbf{k}} \cdot \mathbf{\Omega}_{M_{\mathbf{k}}} \cdot \tilde{\mathbf{A}}_{\mathbf{k}}$, where $\mathbf{\Omega}_{M_{\mathbf{k}}}$ is diagonal. $\mathbf{\Omega}_{M_{\mathbf{k}}}$ is found using the eigenvalue equation

$$(\mathbf{g}\mathbf{M}_{\mathbf{k}}) \cdot \mathbf{S}_{\mathbf{k}} = \mathbf{S}_{\mathbf{k}} \cdot (\mathbf{g}\mathbf{\Omega}_{M_{\mathbf{k}}}) \equiv \mathbf{S}_{\mathbf{k}} \cdot \mathbf{\Omega}_{gM_{\mathbf{k}}}\,. \tag{30}$$

One can show [17] that if $\mathbf{M}_{\mathbf{k}}$ is semidefinite positive then the eigenvectors of $\mathbf{g}\mathbf{M}_{\mathbf{k}}$ come in pairs of opposite eigenvalues and lead to :

$$\mathcal{H} = S(S+1)\frac{E_{\mathsf{clas}}}{S^2} + \frac{S}{2}\sum_{\mathbf{k}}\Big(\omega_1(\mathbf{k}) + \omega_2(\mathbf{k})\Big) + S\sum_{\mathbf{k}}\Big(\omega_1(\mathbf{k})\,\tilde{a}^+_{\mathbf{k},1}\tilde{a}_{\mathbf{k},1} + \omega_2(\mathbf{k})\,\tilde{a}^+_{\mathbf{k},2}\tilde{a}_{\mathbf{k},2}\Big) + \mathcal{O}(\sqrt{S})\,. \tag{31}$$

The ground state of this Hamiltonian is the vacuum of $\tilde{a}_{\mathbf{k},1}, \tilde{a}_{\mathbf{k},2}$, and so the quadratic energy correction is

$$\delta E = \frac{S}{2}\sum_{\mathbf{k}}\Big(\omega_1(\mathbf{k}) + \omega_2(\mathbf{k})\Big)\,. \tag{32}$$

Integrating over the BZ of the lattice, we find the quadratic energy correction as a function of the angle α between the two sublattices. Results are shown in Supplementary Figure 5 (energy per site) for the case of $J_2 = J_3 = 3$ meV and demonstrate that spin waves favor the collinear confingurations $\alpha = 0$ or π.

Supplementary References

[1] Figgen, D., Peterson, K. A., Dolg, M. & Stoll, H. Energy-consistent pseudopotentials and correlation consistent basis sets for the 5d elements Hf–Pt. *J. Chem. Phys.* **130**, 164108 (2009).

[2] Dunning, T. H. Gaussian basis sets for use in correlated molecular calculations. I. The atoms boron through neon and hydrogen. *J. Chem. Phys.* **90**, 1007–1023 (1989).

[3] Fuentealba, P., Preuss, H., Stoll, H. & von Szentpaly, L. A proper account of core-polarization with pseudopotentials: single valence-electron alkali compounds. *Chem. Phys. Lett.* **89**, 418 (1982).

[4] Fink, K., Fink, R. & Staemmler, V. Ab Initio Calculation of the Magnetic Exchange Coupling in Linear Oxo-Bridged Binuclear Complexes of Titanium(III), Vanadium(III), and Chromium(III). *Inorg. Chem.* **33**, 6219 (1994).

[5] van Oosten, A. B., Broer, R. & Nieuwpoort, W. C. Heisenberg exchange enhancement by orbital relaxation in cuprate compounds. *Chem. Phys. Lett.* **257**, 207 (1996).

[6] Broer, R., Hozoi, L. & Nieuwpoort, W. C. Nonorthogonal Approaches to the Study of Magnetic Interactions. *Mol. Phys.* **101**, 233 (2003).

[7] Calzado, C. J., Evangelisti, S. & Maynau, D. Local Orbitals for the Truncation of Inactive Space: Application to Magnetic Systems. *J. Phys. Chem. A* **107**, 7581 (2003).

[8] Katukuri, V. M., Stoll, H., van den Brink, J. & Hozoi, L. *Ab initio* determination of excitation energies and magnetic couplings in correlated quasi-two-dimensional iridates. *Phys. Rev. B* **85**, 220402 (2012).

[9] Katukuri, V. M. *et al.* Mechanism of basal-plane antiferromagnetism in the spin-orbit driven iridate Ba_2IrO_4. *Phys. Rev. X* **4**, 021051 (2014).
[10] Bogdanov, N. A., Katukuri, V. M., Stoll, H., van den Brink, J. & Hozoi, L. Post-perovskite $CaIrO_3$: A $j = 1/2$ quasi-one-dimensional antiferromagnet. *Phys. Rev. B* **85**, 235147 (2012).
[11] Bogdanov, N. A., Maurice, R., Rousochatzakis, I., van den Brink, J. & Hozoi, L. Magnetic State of Pyrochlore $Cd_2Os_2O_7$ Emerging from Strong Competition of Ligand Distortions and Longer-Range Crystalline Anisotropy. *Phys. Rev. Lett.* **110**, 127206 (2013).
[12] Katukuri, V. M. *et al.* Kitaev interactions between $j = 1/2$ moments in honeycomb Na_2IrO_3 are large and ferromagnetic: insights from *ab initio* quantum chemistry calculations. *New J. Phys.* **16**, 013056 (2014).
[13] Pipek, J. & Mezey, P. G. A fast intrinsic localization procedure applicable for abinitio and semiempirical linear combination of atomic orbital wave functions. *J. Chem. Phys.* **90**, 4916 (1989).
[14] Werner, H.-J., Knowles, P. J., Knizia, G., Manby, F. R. & Schütz, M. MOLPRO 2012, see http://www.molpro.net.
[15] Yildirim, T., Harris, A. B., Aharony, A. & Entin-Wohlman, O. Anisotropic spin Hamiltonians due to spin-orbit and Coulomb exchange interactions. *Phys. Rev. B* **52**, 10239 (1995).
[16] Luttinger, J. M. & Tisza, L. Theory of Dipole Interaction in Crystals. *Phys. Rev.* **70**, 954–964 (1946).
[17] Blaizot, J.-P. & Ripka, G. *Quantum Theory of Finite Systems* (Cambridge, MA, 1986).
